# Keratoacanthoma in a Patient With Skin of Color

**DOI:** 10.7759/cureus.82695

**Published:** 2025-04-21

**Authors:** William Stansbury, Micah Pippin, Vincent Pham, Ashley Flowers

**Affiliations:** 1 Family Medicine, Louisiana State University Health Sciences Center, Alexandria, USA; 2 Family Medicine, Rapides Regional Medical Center, Alexandria, USA; 3 Family Medicine, Tulane University School of Medicine, New Orleans, USA; 4 Pathology, Louisiana State University Health Sciences Center, Shreveport, USA

**Keywords:** benign skin tumor, dermatologic neoplasms, keratoacanthoma, skin of color dermatology, squamous cell carcinoma of the skin

## Abstract

Keratoacanthomas are rapidly growing, benign cutaneous neoplasms that can mimic squamous cell carcinoma, presenting diagnostic and management challenges for physicians. The case report investigates a 63-year-old African American female who presented to a family medicine clinic with a one-year history of a progressively enlarging nodule on her right shin. Clinical examination revealed a seven-millimeter dome-shaped, hyperpigmented, well-defined, scaly, rough papule with central umbilication and a keratin plug. Histopathological analysis of an excisional biopsy confirmed the diagnosis of keratoacanthoma with clear margins. Due to the significant overlay in gross appearance and dermoscopic features of keratoacanthomas and malignant squamous cell carcinomas, it is imperative to fully excise similarly presenting lesions and send them for microscopic analysis. Even with histologic examination, keratoacanthomas and squamous cell carcinomas can be challenging to differentiate. Therefore, the priority of contemporary research lies in new methods of distinguishing the two conditions so that the self-limited, benign keratoacanthoma can be managed more conservatively, avoiding invasive removal and associated morbidity. This scholarship is especially pertinent to patients, like the one in this case study, with skin of color who are more likely to experience post-operative complications such as proinflammatory hyperpigmentation, hypertrophic scars, and keloids, but are also at risk of squamous cell carcinoma.

## Introduction

Keratoacanthomas are skin tumors that classically appear as dome-shaped lesions with a centralized cutaneous keratin-plugged nodule on sun-exposed areas [1-2]. Keratoacanthomas originate from the pilosebaceous unit and exhibit a benign cycle of rapid growth, stability, and regression [1-2]. There is much controversy about the nature of keratoacanthomas due to their commonality with squamous cell carcinoma [1-3]. Gross examination and dermoscopy cannot reliably differentiate the two conditions, dictating that an excisional biopsy should be performed for histologic investigation [4]. This case report discusses the general epidemiology, clinical presentation, evaluation, and management of keratoacanthomas, the lesion in the context of skin of color, its complex relationship with squamous cell carcinoma, and current research into reconciling this benign growth with its malignant counterpart.

## Case presentation

A 63-year-old African American female presented to the clinic with concerns about a persistent nodule on her right lower leg. Her medical history was significant for hypertension, osteoarthritis, and anxiety, and her family history was noncontributory. Her medications included amlodipine 10 mg tabs once daily for hypertension, alprazolam 0.5 mg tabs once a day as needed for anxiety, and over-the-counter (OTC) non-steroidal anti-inflammatory drugs (NSAIDs) for chronic arthritic pain. Her surgical history only includes a bilateral tubal ligation for contraception. She had no known drug allergies. The patient first noticed the nodule approximately one year previously and did not recall any associated trauma at that time. The patient stated that the nodule was initially small but had been progressively growing and becoming more raised. She experienced constant itching and localized tenderness of the nodule but had no fever or other systemic symptoms. The patient reported trying multiple over-the-counter treatments, including hydrogen peroxide, calamine lotion, and iodine. None of these treatments provided relief or improvement of the nodule’s appearance or symptoms. Upon further questioning, the patient had accidentally shaved off a part of the nodule a few months prior with a small amount of localized bleeding. Subsequently, the nodule regrew quickly, returning to its previous state and size. The patient also reported experiencing moderate sun exposure due to outdoor activities but denied excessive tanning practices. The patient had a 30-year history of smoking approximately five cigarettes per day. There was no reported history of alcohol or illicit drug use. She had no known drug allergies.

**Figure 1:**
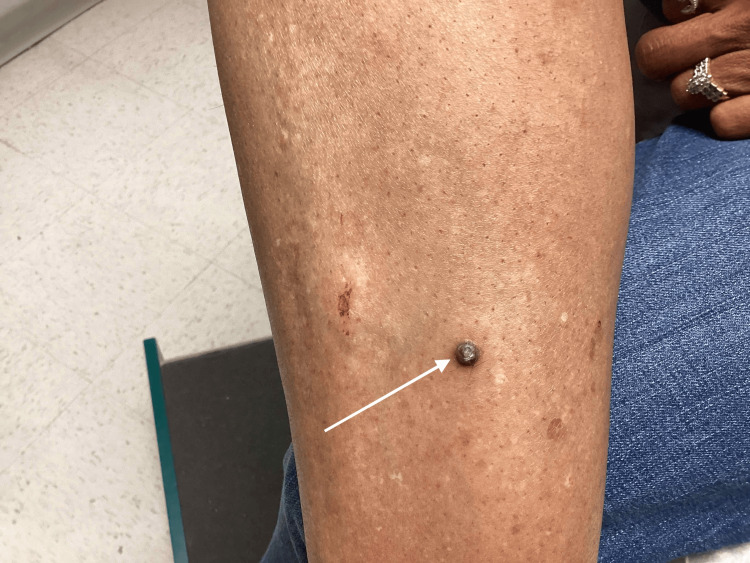
Keratoacanthoma of the right anterior leg

The patient’s vital signs included a blood pressure of 133/89 mmHg, respiratory rate of 20 breaths per minute, heart rate of 82 beats per minute, and a temperature of 98.7 degrees Fahrenheit (37 degrees Celsius). The patient weighed 147 pounds (66.6 kilograms) and had a body mass index (BMI) of 23.02 kilograms per meter squared. The patient appeared well on physical examination and had a Fitzpatrick type five skin tone. She had a seven-millimeter scaly, hyperpigmented, well-defined nodule on her anterior right shin (Figure 1).

**Figure 2:**
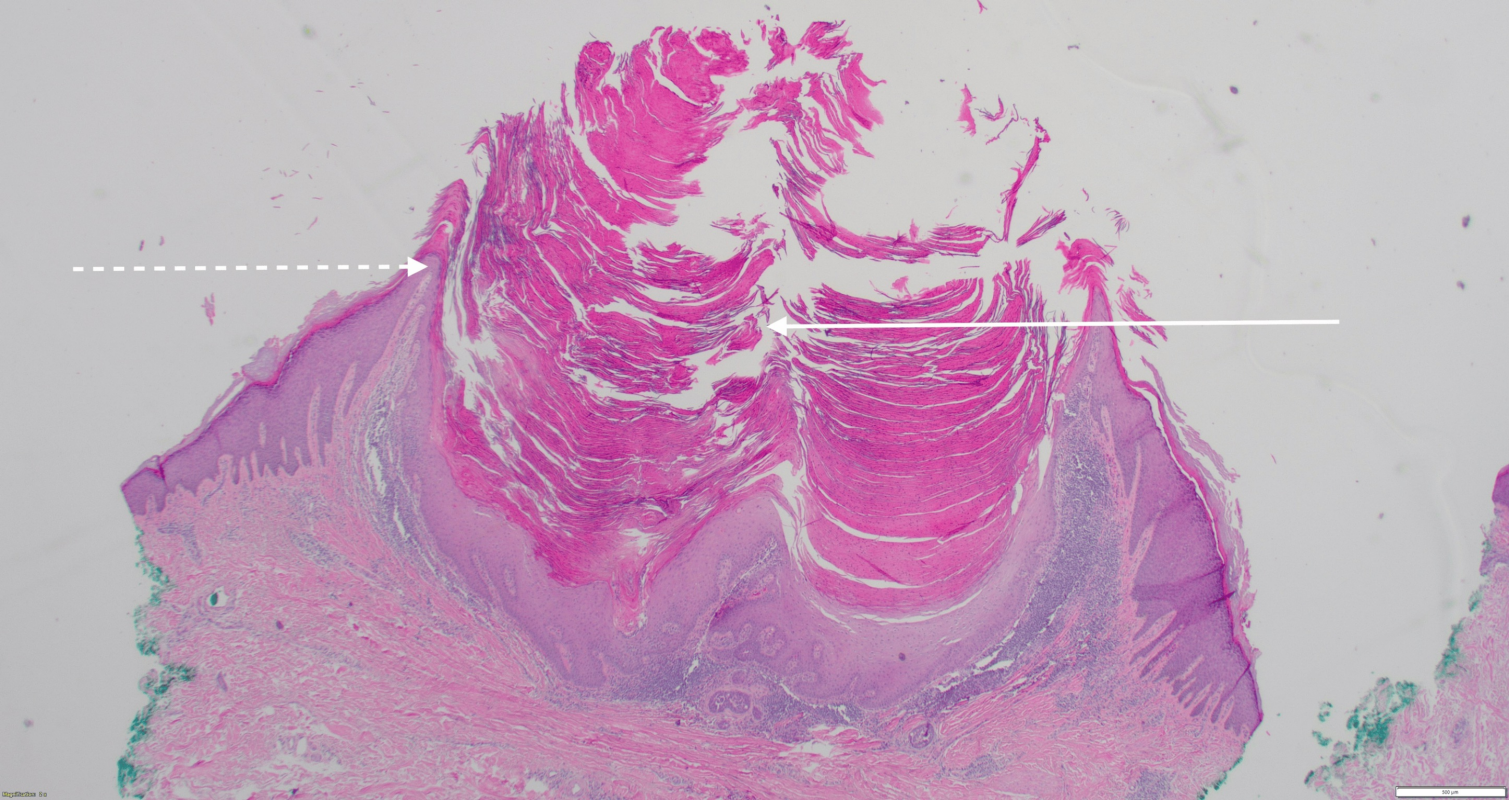
2x magnification hematoxylin and eosin (H&E) stain of a keratoacanthoma demonstrating a symmetric lesion with a centralized keratin plug (solid arrow), epithelial lipping (dashed arrow), and minimal atypia

The mass was solid, non-fluctuant, and had a keratinized umbilicated center. There was no surrounding erythema or telangiectasias. The rest of her examination was unremarkable. The nodule's steady growth over the past year, with an episode of regrowth after being shaven off, prompted concerns that the nodule may be malignant. As such, an eight-millimeter excisional punch biopsy to a depth of four millimeters was performed to assess for malignancy. Histopathology eventually confirmed the nodule as a keratoacanthoma with negative margins (Figure 2).

**Figure 3:**
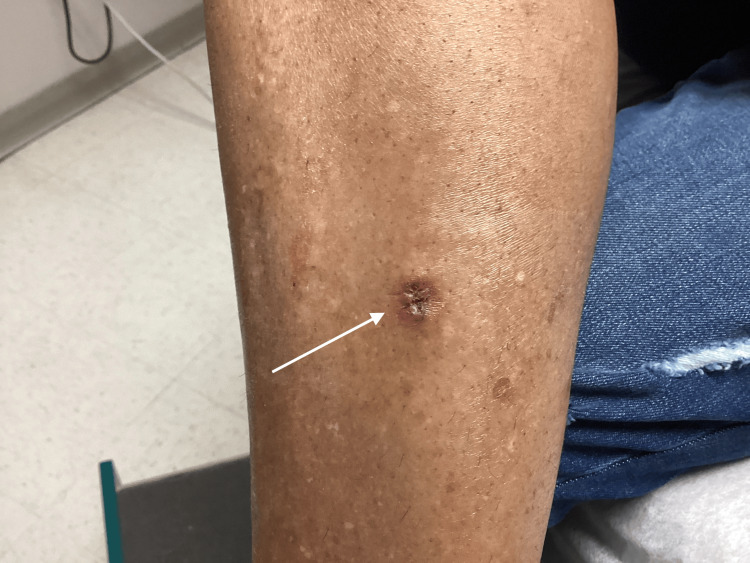
Resolution of the keratoacanthoma 10 days following excision

The patient returned to the clinic 10 days later for a follow-up visit and was healing well at the excision site with no erythema, itching, or drainage (Figure 3).

She was educated on the importance of skin awareness, sunscreen usage, smoking as a risk factor for keratoacanthoma, and the need for cessation, and she was instructed to follow up as needed.

## Discussion

Keratoacanthomas are cutaneous keratin-plugged nodules that spontaneously occur from the pilosebaceous unit, rapidly grow, and then generally regress [1-2]. The peak incidence of solitary keratoacanthoma occurs between 65 and 71 years of age [1]. Risk factors include but are not limited to exposure to ultraviolet light, trauma, human papillomavirus, immunosuppression, and smoking [1]. Although they occur more often in sun-exposed areas like the face, there are several situations, such as the present case, where they may occur in less-exposed areas such as the legs and buttocks [2].

Epidemiologic studies regarding keratoacanthomas are scarce. The first keratoacanthoma population-based incidence study in the United States occurred in Kauai, Hawaii, and was published in 1993 [5]. The study revealed an incidence of 104 cases per 100,000 people with a two-to-one male-to-female ratio and an average patient age of 63.5 years [5]. The largest population study was published in 2020 and reported an Australian incidence of 409 keratoacanthomas per 100,000 patients [6]. This high incidence may be related to increased ultraviolet radiation exposure in the sunny Australian climate [6]. Data shows that most keratoacanthomas occur in people with lightly pigmented skin but may occur in any skin type [3]. There is conflicting data regarding the incidence of keratoacanthomas between sexes. Reports show that the rates range from a similar ratio between males and females to a three times greater risk of developing keratoacanthomas in men [7]. While most keratoacanthomas appear in sun-exposed areas, such as the face, it is important to consider that there may be variations among different sexes. Keratoacanthomas of the hand are found more often in men, compared to the presence of keratoacanthomas of the calf and anterior tibial area being more common in women [7]. Although most cases are sporadic, some reported cases are associated with genetic and other syndromes in the literature, especially when multiple keratoacanthomas manifest rapidly [8-9].

The center of any discussion concerning keratoacanthomas lies in its differential diagnosis. Squamous cell carcinoma is often indistinguishable from keratoacanthomas, having similar gross, dermoscopic, and microscopic appearances and featuring comparable risk factors, age, and gender distributions [4,9-10]. Their interrelation is so significant that there is even controversy about the nomenclature of keratoacanthomas, and they have recently been reclassified as squamous cell carcinoma keratoacanthoma type (SCC-KA) [9-10]. Contemporary scholarship on keratoacanthomas predominantly focuses on the relationship between the two conditions, as this association directs overall management strategies for keratoacanthomas. Although mostly benign, the current recommendation for the treatment of keratoacanthoma-appearing lesions is excision so that histologic evaluation can distinguish the sample from malignant squamous cell carcinoma [2,8]. If an effective method of differentiating keratoacanthoma and squamous cell carcinoma existed, a more conservative strategy could be employed to address the generally benign, self-resolving keratoacanthomas.

Dermoscopic analysis of keratoacanthomas has not been able to distinguish the lesion from squamous cell carcinoma [11-12]. One retrospective analysis identified the core dermoscopic features of both conditions as similar and included white circles, keratin, blood spots, and white structureless zones [11]. The same study and other investigations found that centralized keratin was more common in keratoacanthomas than squamous cell carcinoma; however, this feature was insufficient to distinguish between the two conditions [11-12].

Keratoacanthomas have histologic findings uniquely identified between the proliferation, maturation, and regression stages [2,10]. During the initial phase, there are several ground-glass-appearing islands of laminated keratin that have not yet merged into a centralized keratin plug [10]. No nuclear atypia is present in these structures; however, some may appear on the lesion's periphery [10]. Inflammation may be present, and numerous cell types, including eosinophils, neutrophils, histiocytes, and plasma cells, can be identified [2-3,10]. The maturation stage is when most keratoacanthomas are excised and viewed microscopically [10]. It features the classic findings of a central keratin plug, overhanging epithelial lips, and compact keratinization [2,10]. Pleomorphic cells may be seen on the distant fringes [10]. During regression, the keratin plug recedes, leaving a hollowed-out appearance and perilesional dermal inflammation [10]. While keratoacanthomas and squamous cell carcinomas share similar histologic elements, a 2024 study identified several key distinguishing features. Significant symmetry and epithelial lipping were present in keratoacanthomas but not squamous cell carcinomas [10]. Squamous cell carcinomas demonstrate more cellular atypia than keratoacanthomas and are more invasive, often spreading beyond the sweat gland level [10]. The atypia is more peripherally located in keratoacanthomas while randomly distributed diffusely throughout squamous cell carcinomas [10]. Squamous cell carcinomas exhibit more ulceration and mitosis, and pathognomonic keratin pearls may be present [10].

It is imperative that an adequate tissue sample is obtained during excisional biopsy and that the lesion is removed in its entirety [7,10]. A shave or partial biopsy may miss the identifiable architectural features on which histologic evaluation depends [7,10]. For typical lesions, removal with four-millimeter margins is preferable [10]. Keratoacanthomas located on cosmetically sensitive areas, those larger than two centimeters, or lesions with aggressive features may require Mohs micrographic surgery [2,7,10]. In this case, consultation with an interdisciplinary team composed of a dermatologist or plastic surgeon would be advised. Several nonsurgical interventions, such as the use of topical preparations and intralesional injections, have been investigated but lack significant evidence as of yet [2].

New research has focused on methods to distinguish keratoacanthomas from squamous cell carcinomas when differentiation through traditional microscopic analysis is impossible. While there are currently no immunohistochemical stains capable of reliably discriminating between keratoacanthomas and squamous cell carcinoma, investigators are exploring new staining techniques and the use of genetic, molecular, and immune markers [10]. One 2023 study utilized spatial omics technology to characterize the tumor microenvironment of keratoacanthomas and squamous cell carcinomas to distinguish the two and drive the development of future diagnostic markers [13].

While keratoacanthomas are less common in skin of color, they remain an important clinical entity for individuals with darker complexions, such as the patient presented in this case. Keratoacanthoma's similarity to squamous cell carcinoma is of particular importance in Black patients as squamous cell carcinoma is the most prevalent dermatologic malignancy in this population and carries a higher mortality rate than in white patients [14]. It is, therefore, paramount to confirm that a presumptive keratoacanthoma lesion is excised to exclude squamous cell carcinoma from the differential. While keratoacanthomas' morphology and general appearance are comparable in patients with fair and dark skin, erythema is less likely to be visualized in dark skin, and inflammatory hyperpigmentation is more prevalent [15]. The field of dermoscopy is in relative infancy for utilization in patients with dark skin compared to those with lighter skin. Less data is available; however, keratoacanthomas appear to have similar dermoscopic findings in skin of color [15-16]. Still, increased melanin pigmentation in dark skin can obscure traditional dermoscopic variables [15-16]. In the context of darker skin types, research into methods of distinguishing between keratoacanthomas and squamous cell carcinomas is especially vital as surgical excision, currently required due to the condition's ambiguity, is more likely to result in postoperative complications such as post-inflammatory hyperpigmentation, hypertrophic scars, and keloids [14].

## Conclusions

This case highlights the importance of considering keratoacanthoma in the differential diagnosis of rapidly growing cutaneous nodules, particularly in patients with darker skin tones, where its occurrence is less commonly documented. Despite its benign nature, keratoacanthoma can mimic squamous cell carcinoma, necessitating a thorough clinical evaluation and histopathological confirmation to ensure appropriate management. The successful excision of the lesion in this patient underscores the effectiveness of early recognition and surgical intervention in preventing potential complications. Physicians should remain vigilant for atypical presentations of keratoacanthoma and educate patients on the importance of skin awareness to facilitate timely diagnosis and treatment. Further studies are warranted to explore the underlying factors influencing keratoacanthoma development and progression, particularly in underrepresented populations, and to develop reliable means of differentiating keratoacanthomas from squamous cell carcinoma.
